# Cutaneous Metastasis Mimicking a Pyogenic Granuloma Revealing Renal Cell Carcinoma

**DOI:** 10.7759/cureus.81232

**Published:** 2025-03-26

**Authors:** Meryem Soughi, Riham Alheyasat, Hanane Baybay, Layla Tahiri Elousrouti, FatimaZahra Mernissi

**Affiliations:** 1 Department of Dermatology, University Hospital Hassan II, Unités de Recherche Labellisées (URL) Centre National pour la Recherche Scientifique et Technique (CNRST) N15, Fez, MAR; 2 Human Pathology, Biomedicine and Environment Laboratory, Faculty of Medicine, Pharmacy and Dental Medicine, Sidi Mohamed ben Abdellah University, Fez, MAR; 3 Department of Dermatology, University Hospital Hassan II, Fez, MAR; 4 Department of Pathology, University Hospital Hassan II, Fez, MAR

**Keywords:** cutaneous metastasis, dermoscopy, kidney cancers, pyogenic granuloma, renal cell carcinoma

## Abstract

Renal cell carcinoma (RCC) is a malignancy that can, in rare cases, present initially with cutaneous metastases, often reflecting an advanced stage. We report the case of a 71-year-old asymptomatic patient who presented with a gradually enlarging, raised lesion on the face over two years, clinically resembling a pyogenic granuloma. An excisional biopsy confirmed the diagnosis of cutaneous metastasis from clear cell RCC. Further staging investigations revealed a mass in the upper pole of the left renal cortex, a suspicious lesion in the lower pole, and bilateral pulmonary nodules, suggesting metastatic spread. The patient was started on sunitinib, resulting in both clinical and radiological improvement.

## Introduction

Renal cell carcinoma (RCC) represents 2-3% of adult solid malignancies, with a higher prevalence in males than in females [1,2]. The most common type of RCC is clear cell carcinoma, which accounts for up to 60% of cases [3]. The most frequent sites of metastatic disease include regional lymph nodes, lungs, and bones [4]. However, cutaneous metastases are rare, with an incidence of 3.4% among kidney cancers, often indicating an advanced stage of the disease [3]. In this context, we report a case of cutaneous metastasis mimicking a pyogenic granuloma, which led to the diagnosis of metastatic renal cell carcinoma.

## Case presentation

**Figure 1:**
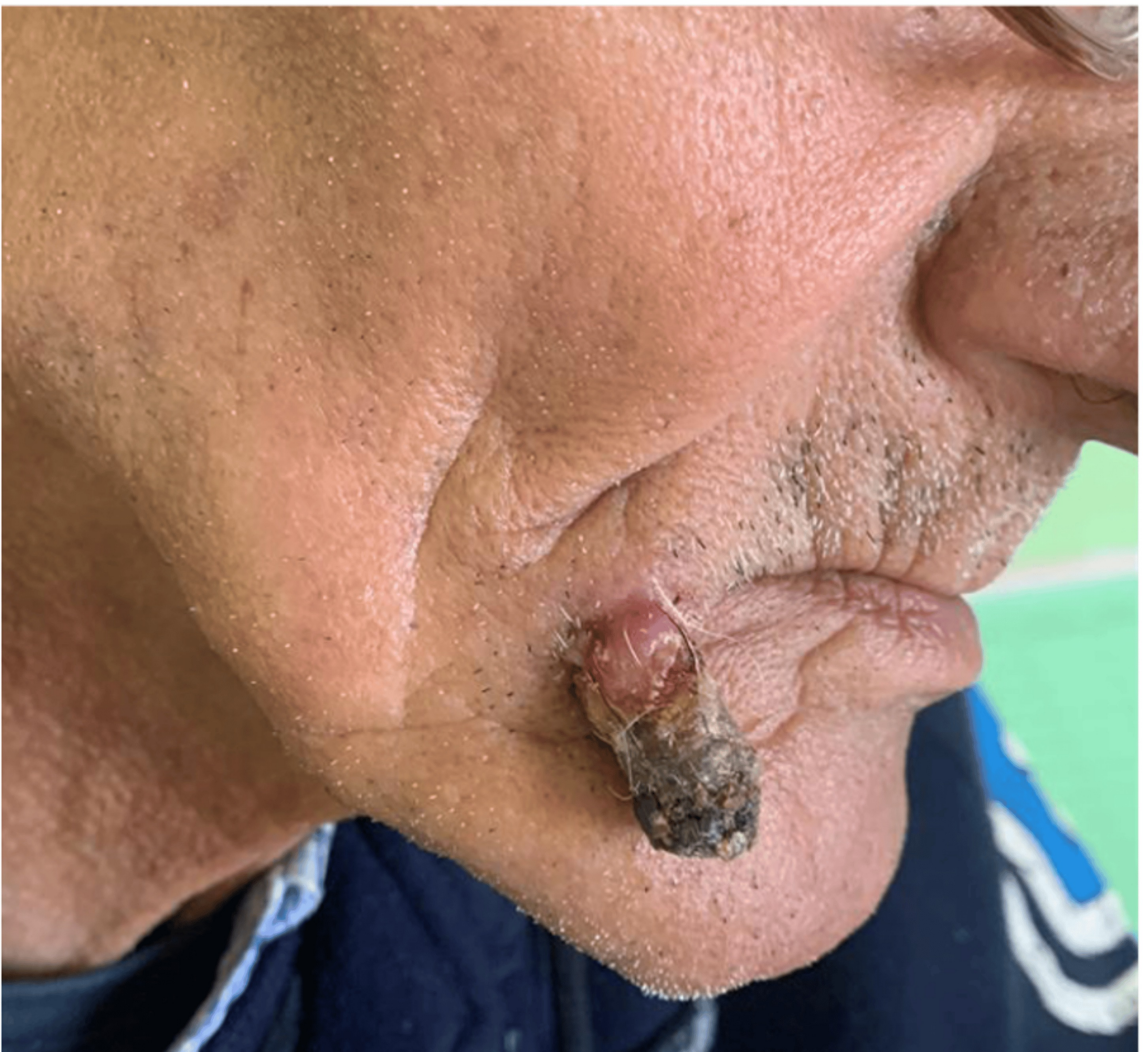
Clinical exam: nodule covered by hemorrhagic crusts, non-bleeding upon palpation, sessile at the base, non-infiltrative, measuring 2 cm at its largest dimension, located beneath the right labial commissure

**Figure 2:**
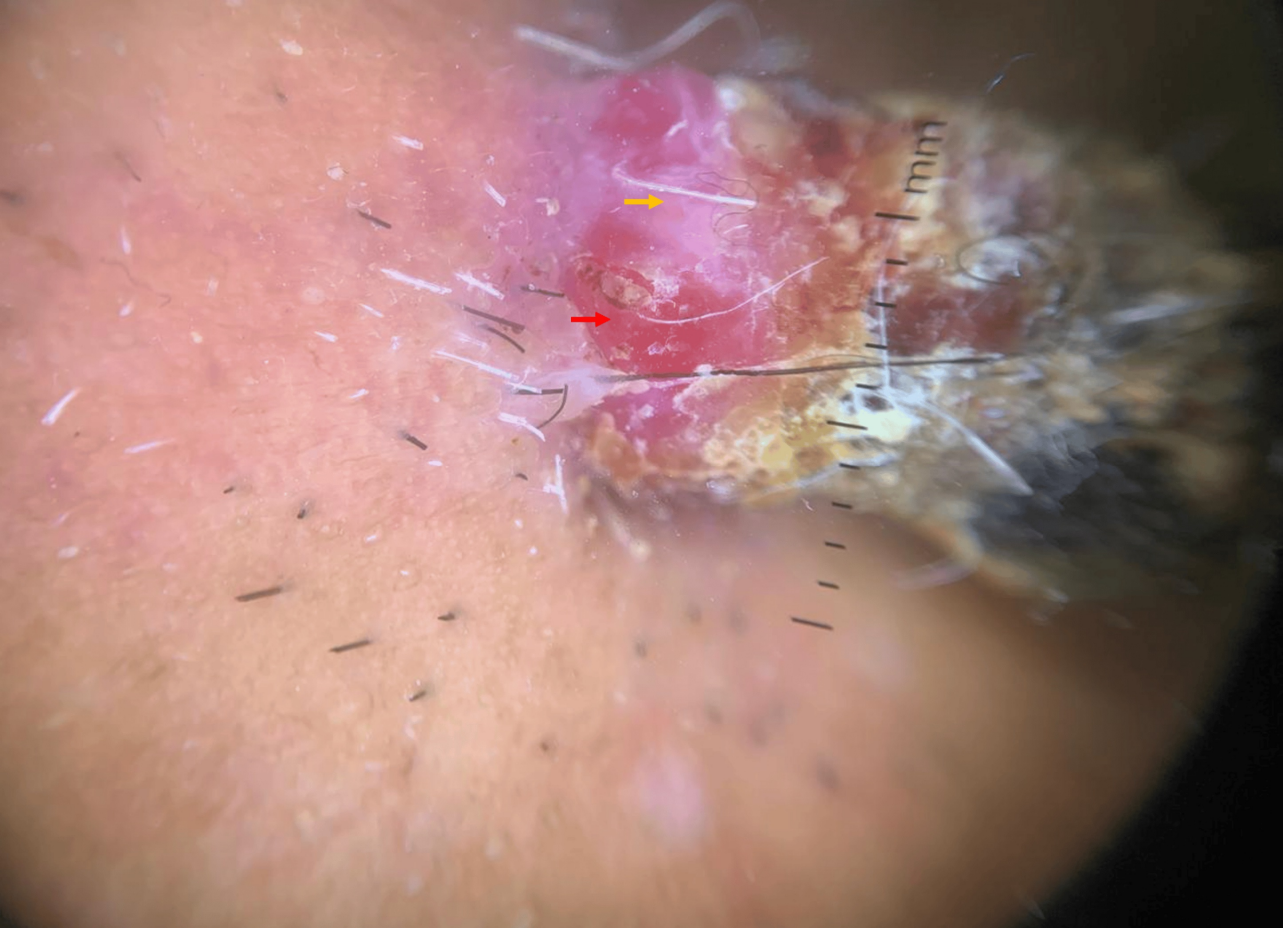
Dermoscopic aspect: erythematous background (red arrow) and septa (yellow arrow)

**Figure 3:**
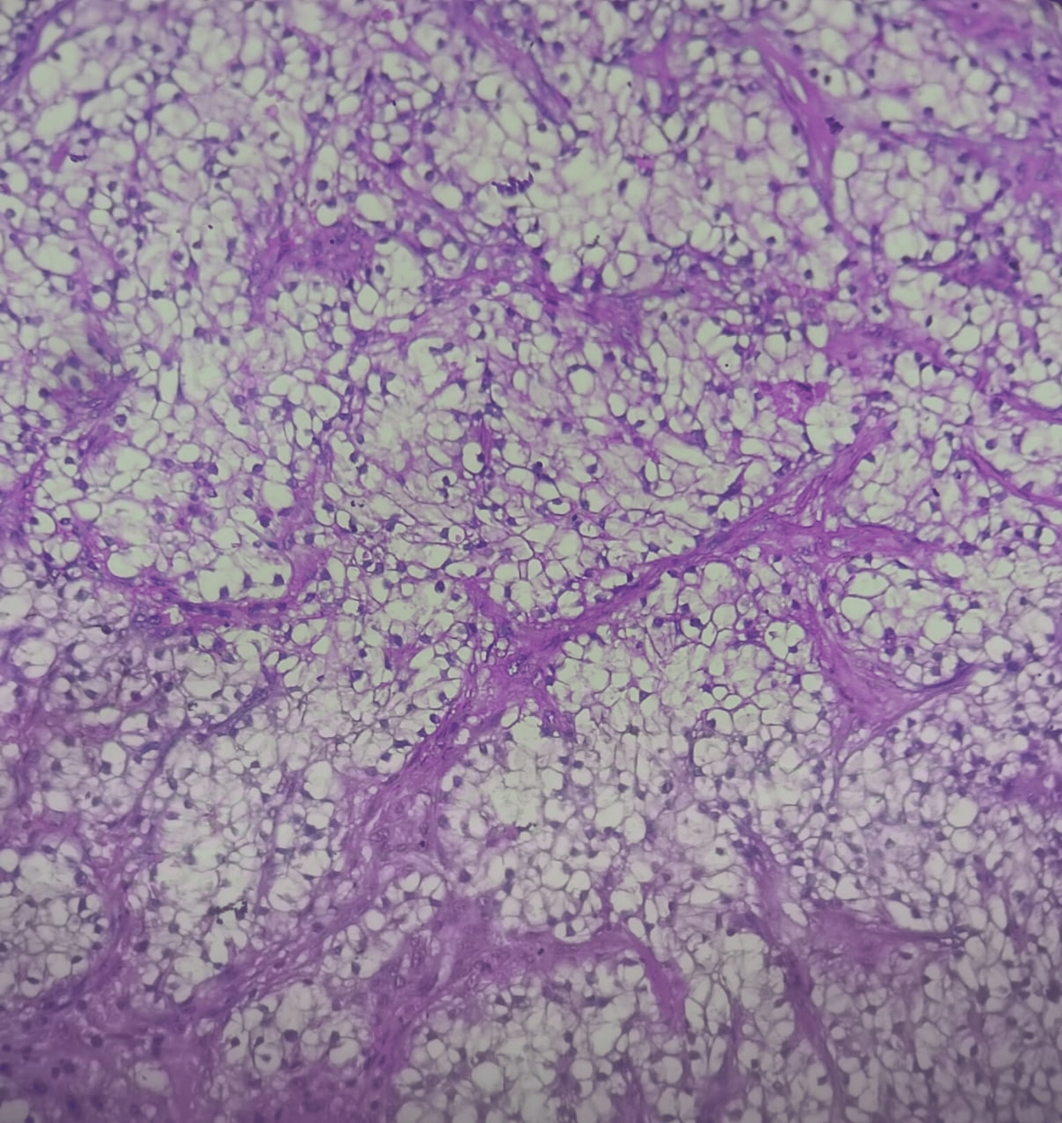
Histological features observed at 200× magnification with H&E staining include tumor proliferation composed of cohesive clear cells arranged into nests and lobules, separated by a fibrous stroma

**Figure 4:**
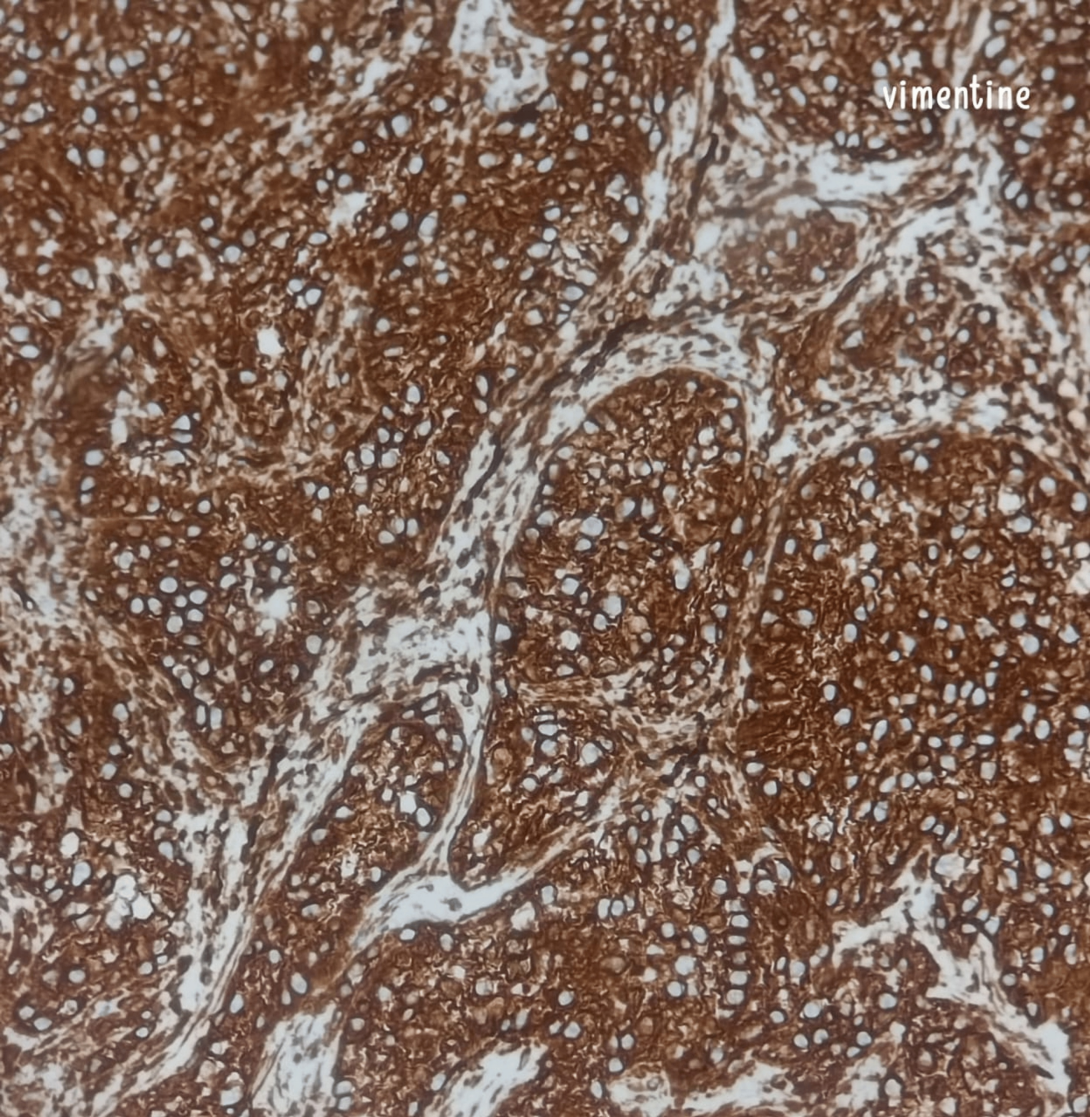
Immunohistochemical staining at 200x magnification shows strong expression of vimentin

**Figure 5:**
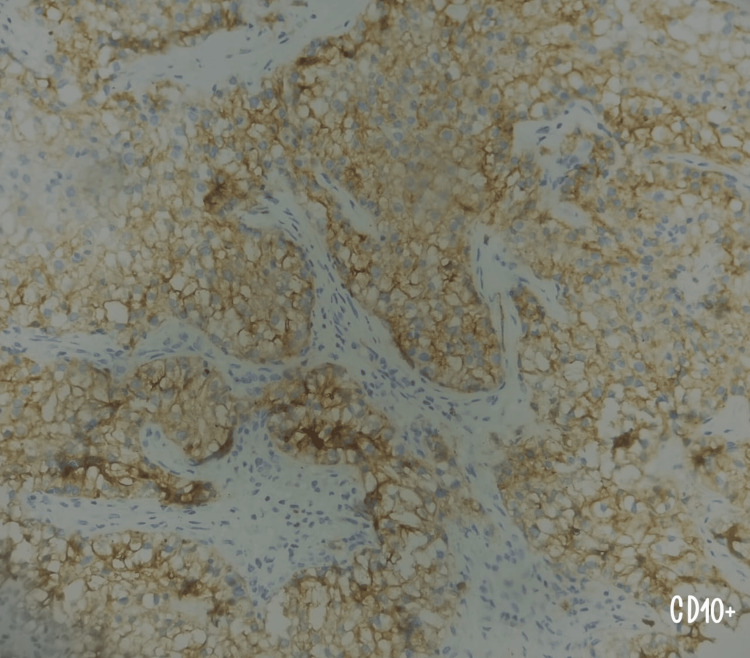
Immunohistochemical staining at 200x magnification shows strong expression of CD10

**Figure 6:**
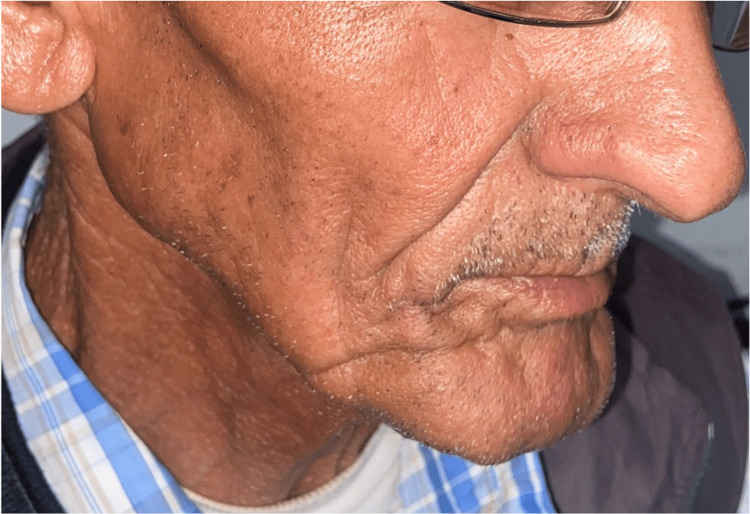
Good healing after surgical excision of skin metastases

**Figure 7:**
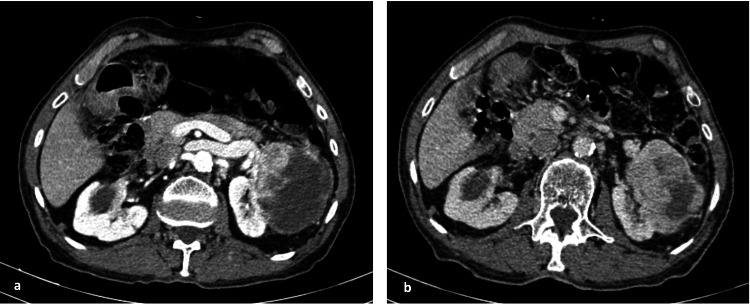
CT scan imaging showing significant tumor regression a: before treatment; b: after six months of treatment

A 71-year-old male patient with no significant medical history presented with a progressively enlarging raised lesion on his face for the past two years. Dermatological examination revealed an erythematous nodule covered by hemorrhagic crusts, non-bleeding upon palpation, sessile at the base, non-infiltrative, measuring 2 cm at its largest dimension, located beneath the right labial commissure (Figure 1). Dermoscopy showed an erythematous background, whitish structures, and septa (Figure 2). An excisional biopsy was performed, revealing a tumor proliferation with clear cells (Figure 3), raising the possibility of either a primary cutaneous tumor, such as squamous cell carcinoma with a clear cell variant, or an adnexal carcinoma such as sebaceous carcinoma. However, a secondary metastasis from renal clear cell carcinoma could not be excluded. An immunohistochemical analysis demonstrated strong expression of vimentin (Figure 4) and CD10 (Figure 5), with no expression of CK7 and CK5/6, confirming the diagnosis of cutaneous metastasis from renal cell carcinoma (clear cell type). Staging investigations revealed a mass in the left renal cortex at the upper pole, consistent with clear cell renal carcinoma, a suspicious lesion in the lower pole of the left kidney, as well as bilateral diffuse pulmonary parenchymal nodules and micronodules suggestive of secondary metastases. The patient was treated with sunitinib, and clinical and radiological improvements were observed (Figure 6) (Figure 7).

## Discussion

Renal cell carcinomas (RCC) account for 2-3% of malignant tumors and are most commonly seen in adults, typically between the 5th and 7th decades of life [4]. The classic triad of RCC consists of a palpable mass, hematuria, and back pain; however, only 10% of patients present with all three symptoms [5]. In contrast, our patient, who was elderly, was asymptomatic at the time of tumor discovery, despite the tumor already having progressed to a metastatic stage. Cutaneous metastases from renal tumors generally occur as hematogenous emboli resulting from tumor invasion of the renal vein [4]. The skin is the seventh most common site of metastatic involvement [4]. Approximately 80% of cutaneous lesions are detected after the primary renal neoplasm is diagnosed, while 10-20% of patients present with skin lesions before the primary malignancy is identified. These cutaneous lesions typically present as subcutaneous, well-circumscribed nodules or infiltrated plaques. They may appear as rapidly growing, nodular, round, or oval lesions, with colors ranging from normal skin tones to red-purple shades [6,7]. The most common locations for these lesions are the face and scalp [5,8], as seen in our patient, who had a lesion near the right labial commissure with erythematous coloration. Dermoscopy revealed features suggestive of pyogenic granulomas, traumatic hemangiomas, or Kaposi’s sarcoma. The clinical diagnosis was challenging, especially since the patient was asymptomatic despite the presence of pulmonary metastases at the time of diagnosis. This led to the indication for treatment with sunitinib. Indeed, the treatment of metastatic renal adenocarcinoma typically involves a combination of surgical treatment (radical nephrectomy) and angiogenesis/multikinase inhibitors such as sunitinib or sorafenib [9].

## Conclusions

This case is of particular interest for two reasons: it presents a rare cutaneous metastasis revealing renal carcinoma and emphasizes the distinctive clinical features of the lesion. It also highlights the importance of considering alternative diagnoses when encountering a lesion resembling a botryomycoma in an elderly patient.
